# SHP2 acts both upstream and downstream of multiple receptor tyrosine kinases to promote basal-like and triple-negative breast cancer

**DOI:** 10.1186/s13058-015-0659-z

**Published:** 2016-01-04

**Authors:** Fatimah Matalkah, Elisha Martin, Hua Zhao, Yehenew M. Agazie

**Affiliations:** Department of Biochemistry, West Virginia University, Morgantown, WV 26506 USA; The Marry Babb Randolph Cancer Center, School of Medicine, West Virginia University, Morgantown, WV 26506 USA

**Keywords:** Breast cancer, SHP2, EGFR, FGFR, c-Met

## Abstract

**Introduction:**

Dysregulated receptor tyrosine kinase (RTK) signaling is a common occurrence in basal-like and triple-negative breast cancer (BTBC). As a result, RTK-targeting therapies have been initiated but proved difficult, mainly owing to the multiplicity of dysregulated RTKs. Hence, targeting master regulators of RTK signaling might alleviate this obstacle. Before that, however, defining the mechanism of such molecules is required. In this report, we show that the Src homology phosphotyrosyl phosphatase 2 (SHP2) is a master regulator of RTK expression and signaling in BTBC.

**Methods:**

Xenograft tumor growth studies were used to determine the effect of SHP2 inhibition on tumorigenesis and/or metastasis. Cell proliferation rate, anchorage-independent growth, mammosphere formation, and ALDEFLUOR assays were used to compare the relative functional importance of SHP2 and the epidermal growth factor receptor (EGFR) in BTBC cells. Immunohistochemistry and immunofluorescence analyses were used to determine the state of SHP2 and EGFR coexpression in BTBC. Analysis of mitogenic and cell survival signaling was performed to show SHP2’s role in signaling by multiple RTKs.

**Results:**

Inhibition of SHP2 in BTBC cells suppresses their tumorigenic and metastatic properties. Because EGFR is the most commonly dysregulated RTK in BTBC, we first tested the effect of SHP2 inhibition on EGFR signaling and found that SHP2 is important not only for mediation of the Ras/extracellular signal-regulated kinase and the phosphatidyl inositol 3-kinase/Akt signaling pathways but also for the expression of the receptor itself. The existence of a tight association between SHP2 and EGFR expression in tumors and cell lines further suggested the importance of SHP2 in EGFR expression. Comparison of relative biological significance showed the superiority of SHP2 inhibition over that of EGFR, suggesting the existence of additional RTKs regulated by SHP2. Indeed, we found that the expression as well as the signaling efficiency of c-Met and fibroblast growth factor receptor 1, two other RTKs known to be dysregulated in BTBC, are SHP2-dependent. To our knowledge, this is the first demonstration of SHP2 acting both upstream and downstream of RTKs to promote signaling.

**Conclusions:**

SHP2 upregulates the expression and signaling of multiple RTKs to promote BTBC. These findings provide a mechanistic explanation for the superiority of SHP2 inhibition in BTBC.

**Electronic supplementary material:**

The online version of this article (doi:10.1186/s13058-015-0659-z) contains supplementary material, which is available to authorized users.

## Introduction

Of all breast cancer subtypes, basal-like and triple-negative breast cancer (BTBC) is the most aggressive form, causing disproportionately high mortality in women [1, 2]. The lack of targeted therapy, together with the multiplicity of dysregulated molecules, is the major factor that exacerbates poor clinical outcomes. Dysregulation of multiple receptor tyrosine kinases (RTKs), including epidermal growth factor receptor (EGFR), fibroblast growth factor receptor 1 (FGFR1), and hepatocyte growth factor receptor (HGFR; also called *c-Met*) [3–5], is one of the major molecular aberrations in BTBC. Of these, EGFR is the most commonly dysregulated RTK in BTBC.

EGFR belongs to a family of RTKs that includes EGFR itself, EGFR2, EGFR3, and EGFR4. The human counterparts are called human EGFR1–EGFR4 (or HER1–HER4). All members are composed of an extracellular region, a single-pass transmembrane region, and a cytoplasmic region. With the exception of human epidermal growth factor receptor 2 (HER2), all members are activated by ligand binding to the extracellular domain, and all except HER3 have a functional tyrosine kinase domain in the cytoplasmic regions. Activation leads to homo- or heterodimerization at the cell surface and transphosphorylation in the C-terminal tail in the cytoplasm [6–9]. Phosphorylated Tyr residues serve as binding sites for Src homology 2 (SH2) and phosphotyrosine binding domain–containing signaling proteins [10].

Because EGFR is a potent activator of mitogenic and cell survival signaling, its overexpression in cancer is suggested to contribute to tumorigenesis [11], on the basis of which anti-EGFR therapies are being sought [12, 13]. A large body of literature shows that Src homology phosphotyrosyl phosphatase 2 (SHP2) is an essential downstream effector of EGFR signaling [14–17]. Therefore, it is entirely possible that dysregulated EGFR signaling in BTBC also is SHP2-dependent. The upregulated expression of SHP2 in breast cancer, including BTBC [18, 19], and its positive role in breast cancer cell transformation [20, 21] provide supporting evidence for this possibility. SHP2 is a cytoplasmic protein with two SH2 domains in the N terminal region and a phosphotyrosyl phosphatase domain in the C-terminal region [15]. The PTPase function of SHP2 is activated by interaction with Tyr-phosphorylated receptors and adaptor proteins through its SH2 domains [22, 23]. Therefore, dysregulated tyrosine kinase signaling in BTBC can superactivate SHP2.

We recently demonstrated that SHP2 promotes the transformation and invasive property of BTBC cells [24], but its role on BTBC tumorigenesis in vivo was not determined. In addition, the molecular mechanism of SHP2 in promoting BTBC is unknown. In this report, we show, for the first time to our knowledge, that SHP2 promotes BTBC tumorigenesis by mediating not only downstream RTK signaling but also receptor expression.

## Materials and methods

### Cells, cell cultures, and reagents

MDA-MB-231, MDA-MB-468, and MCF-10A cells were purchased from the American Type Culture Collection (Manassas, VA, USA), and the mouse embryonic fibroblasts (MEFs) were received from Dr. Steven Frisch (West Virginia University). With regard to human mammary luminal epithelial (HMLE) cells, only cell lysates received from Dr. Alexey Ivanov (West Virginia University) were used. The conditions for cell growth were described previously [21, 25]. Antibodies used in the study were anti-EGFR (610017) and anti-SHP2 (610822) from BD Biosciences (San Jose, CA, USA); anti-CBL (sc-1651), anti-pan-extracellular signal-regulated kinase 2 (anti-pan-ERK2; sc81457), and anti-ubiquitin (sc271289) from Santa Cruz Biotechnology (Santa Cruz, CA, USA); anti-β-actin (A5441) from Sigma-Aldrich (St. Louis, MO, USA); and anti-phospho-ERK1/2 (9101S), anti-phospho-Akt (9271S), and anti-pan-Akt (11E7) from Cell Signaling Technology (Danvers, MA, USA).

### Silencing SHP2 and EGFR expression

We described silencing SHP2 expression in the MDA-MB-231 and the MDA-MB-468 cells previously [24, 25]. For silencing EGFR, two short hairpin RNA (shRNA) sequences described previously [26] were custom-synthesized (Integrated DNA Technologies, Coralville, IA, USA) and ligated into the lentivirus vector described for SHP2 shRNA constructs. Lentivirus particle production and target cell infections are described in our previous reports [24, 25].

### Induction of tumor growth by intramammary transplantation

Female non-obese diabetic/severe combined immunodeficiency (NOD/SCID) mice were purchased from The Jackson Laboratory (Bar Harbor, ME, USA). Approximately 2 × 10^6^ MDA-MB-231 or 10^6^ MDA-MB-468 cells expressing control or SHP2 shRNA were mixed in a 1:1 ratio with Matrigel (BD Biosciences) and injected into the mammary fat pad of each mouse. Because shRNA-2 (sh-2) was more efficient in silencing SHP2 expression, we used these cells for inducing tumorigenesis. We used 13 mice for the MDA-MB-231–derived cells (6 for control and 7 for shRNA) and 12 for the MDA-MB-468 cells (6 for control and 6 for shRNA) in these studies. Tumor growth was monitored by measuring tumor volume with a caliper. The length (L) and the width (W) were measured directly, and the height was estimated by calculating the average of the two measurements. Hence, the formula L × W × (L + W)/2 was used to obtain tumor volume in cubic millimeters. Tumors, lungs, and liver tissues were harvested after the mice were killed. All experiments were performed according to the West Virginia University Animal Care and Use Committee guidelines.

### Cell lysates, immunoblotting, and immunoprecipitation analyses

Cell lysates were prepared in a buffer described previously [24, 25]. Preparation of samples for total cell lysate analyses, immunoprecipitation analyses, and electrophoretic separation and immunoblotting analyses were conducted as described previously [24, 25].

### Tetramethylrhodamine -labeled EGF fluorescence studies on dynamics of EGFR degradation

Fluorescence studies to examine the dynamics of epidermal growth factor (EGF)-induced EGFR degradation were conducted as described previously by us and others [27, 28]. The only additional steps in the present study were chloroquine treatment of cells for 6 h before chilling and treatment with tetramethylrhodamine-labeled EGF. Fluorescence images were captured using an Olympus IX71 microscope with an attached DP30BW digital camera and MicroSuite Basic Edition software (Olympus America, Melville, NY, USA).

### Quantitative real-time polymerase chain reaction

EGFR messenger RNA (mRNA) level was determined by quantitative reverse transcriptase–polymerase chain reaction (qRT-PCR) using iScript reverse transcription Supermix and iQ SYBR Green Supermix according to the manufacturer’s protocol (Bio-Rad Laboratories, Hercules, CA, USA). The forward primer used was 5′-CCAAAGGTCATCAACTCCCAA-3′, and the reverse primer was 5′-AAGTGCCTATCAAGTGGATGG-3′. For glyceraldehyde-3-phosphate dehydrogenase (GAPDH), the forward primer used was 5′-ACAGCCTCAAGATCATCAGCAATG-3′, and the reverse primer was 5′-TGTGGTCATGAGTCCTTCCACGATAG-3′. The EGFR mRNA expression level was corrected against GAPDH mRNA in both cell lines.

### Immunohistochemistry

The breast tumor specimens, which were diagnosed as BTBC at the Ruby Memorial Hospital of West Virginia University, were obtained from the tissue bank of the Department of Pathology, School of Medicine, West Virginia University. The tumor samples were provided with internal codes of the tissue bank; we did not have any access to patient identifiers. The tumor sections used for immunohistochemistry (IHC) were prepared and processed using a standard protocol. The SHP2 slides were scored as described previously [19], and the Dako staining and visualization method (code 7298) was used for EGFR slides (Dako, Carpinteria, CA, USA).

### Immunofluorescence

Immunofluorescence (IF) of tissue sections was conducted as described previously [19]. The anti-SHP2 (sc-7384; Santa Cruz Biotechnology) and anti-EGFR (E1157; Sigma-Aldrich) antibodies were used for IF staining, and images were collected using an Olympus IX71 microscope equipped with a DP30BW digital camera and MicroSuite software.

### Determining cell proliferation rate

The cell growth rate was determined by direct counting using randomly collected microscopic pictures. Cells were thinly seeded in 100-mm cultures dishes, and pictures were collected at a 4× lens objective in 10 random fields immediately after attachment, and then every 24 h thereafter for a total of 3 days. The average of cells in fixed quadrants in each image was used for comparison of cell proliferation rates. The growth rate was determined by dividing the averages at each time point by the average of the initial time point.

### Anchorage-independent growth assay

Cell transformation was determined by anchorage-independent growth in soft agar as described previously [21, 29]. Colony formation was monitored by visualization under a microscope, and pictures were taken using an Olympus IX71 microscope equipped with a DP30BW digital camera.

### Mammosphere formation assay

We used a modified version of a previously described protocol [30] in this study. Briefly, cells were seeded in ultra-low attachment culture plates (approximately 5 × 10^5^ cells per 6-cm plate) in a medium containing serum-free Dulbecco’s modified Eagle’s medium, 1 μg/ml hydrocortisone, 10 μg/ml insulin, 10 ng/ml EGF, 10 ng/ml fibroblast growth factor (FGF), 5 ng/ml heparin, and Gibco B-27 supplement (Life Technologies/Thermo Fisher Scientific, Grand Island, NY, USA). After 10 days, primary mammospheres were collected by centrifugation at 1000 rpm and dissociated to single cells by trypsination and pipetting. Dissociated cells were recultured under the same conditions to observe the effects of SHP2 silencing on secondary mammosphere-forming efficiency. Pictures were collected after 10 days in both cases.

### ALDEFLUOR assay

The proportion of aldehyde dehydrogenase 1 (ALDH1)-high cells in the control and SHP2-silenced BTBC cells was determined using the ALDEFLUOR assay kit (catalog number 01700; STEMCELL Technologies, Vancouver, BC, Canada) according to the manufacturer’s protocol. Cells were then sorted using FACSDiva version 6.1.3 (BD Biosciences) to determine the proportion of cells with high ALDH1 activity.

## Results

### Inhibition of SHP2 in BTBC cells suppresses tumorigenesis and metastasis

**Fig. 1:**
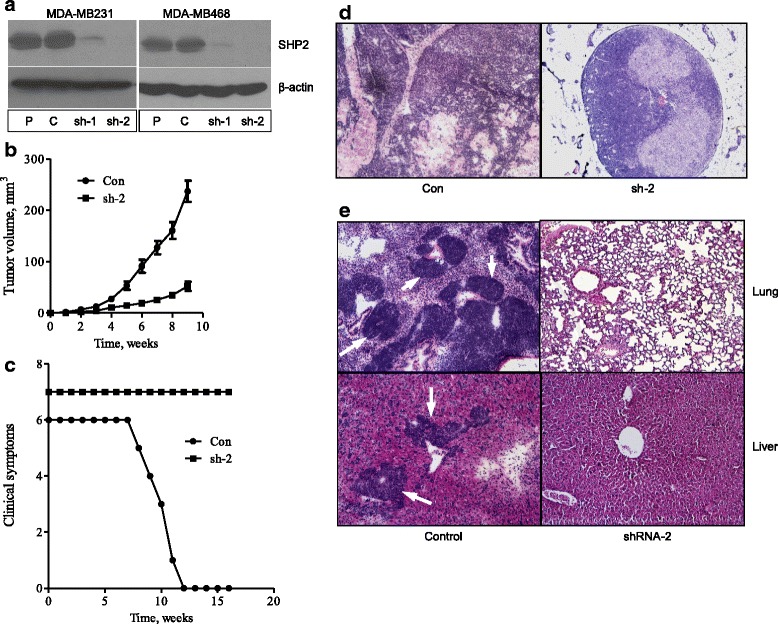
In vivo tumorigenesis studies. **a** Analysis of Src homology phosphotyrosyl phosphatase 2 (SHP2) expression in parental (P), control (C), shRNA-1 (sh-1), and shRNA-2 (sh-2) cells derived from MDA-MB-231 and MDA-MB-468 cells. **b** Tumor growth rate initiated by transplantation of control (Con) and SHP2-silenced (sh-2) cells derived from the MDA-MB-231 cell line. Data shown are mean ± standard error of the mean of tumor volume collected over a period of 9 weeks from six mice in each group. **c** Effect of SHP2 silencing on survival of mice that received transplanted Con and sh-2 cells. **d** Hematoxylin and eosin (H & E)–stained mammary gland tumor sections harvested from mice that received transplanted control and sh-2 MDA-MB-231 cells. **e** H & E staining of lung and liver sections harvested from mice that received transplanted control and sh-2 MDA-MB-231 cells

The role of SHP2 on BTBC tumorigenesis was studied by intramammary transplantation of control and SHP2-silenced BTBC cells in NOD/SCID mice. MDA-MB-231 and MDA-MB-468 cells, which are known to be basal-like and triple-negative and to harbor multiple genetic aberrations commonly discovered in BTBC [31–33], were used for these studies. The expression of SHP2 was silenced with two independent shRNAs (sh-1 and sh-2) that were previously shown to be highly specific and devoid of any off-target effects [24, 25, 34]. Immunoblotting data demonstrating the efficiency of SHP2 silencing is shown in Fig. 1a. Because sh-2 showed efficient silencing, we chose these cells for in vivo studies.

Tumors induced by MDA-MB-231 cells grew very slowly, reaching approximately 250 mm^3^ in the controls and 50 mm^3^ in the shRNA cells within about 9 weeks (Fig. 1b). Around this time, the control mice manifested clinical symptoms such as shortened breathing and reduced demeanor. These mice were killed in succession as they developed these symptoms. However, none of the shRNA mice developed such symptoms, even by 16 weeks (Fig. 1c). These findings suggest that inhibition of SHP2 suppresses tumorigenesis and confers survival.

Hematoxylin and eosin (H & E)–stained sections of mammary tumors showed invasive carcinoma in the controls and connective tissue–encapsulated small tumors in the shRNA mice (Fig. 1d). Similar analysis of lung and liver sections showed multiple pulmonary and liver metastases in the controls and no such lesions in the shRNA mice (Fig. 1e). These results demonstrate that silencing SHP2 expression in the MDA-MB-231 cells suppressed tumorigenesis and/or metastasis.

Tumors induced by the control MDA-MB-468 cells grew relatively quickly, reaching approximately 2300 mm^3^ over the course of 7 weeks, but SHP2-silenced tumors did not reach even 500 mm^3^ during this time (Additional file 1: Figure S1a). Clinically, tumor burden was the major issue with this cell line. As a result, we used an average tumor size of 2000 mm^3^ as an endpoint to determine survival. We found that tumors in the control mice reached this point within 7 weeks, while none in the shRNA mice did even by 13 weeks. Analysis of H & E–stained tissue sections showed only local invasiveness in the mammary glands of control mice (Additional file 1: Figure S1b and c). Regardless of this, inhibition of SHP2 suppressed the tumorigenic property of this cell line as well.

### SHP2 promotes sustained EGFR expression and signaling in BTBC cells

**Fig. 2:**
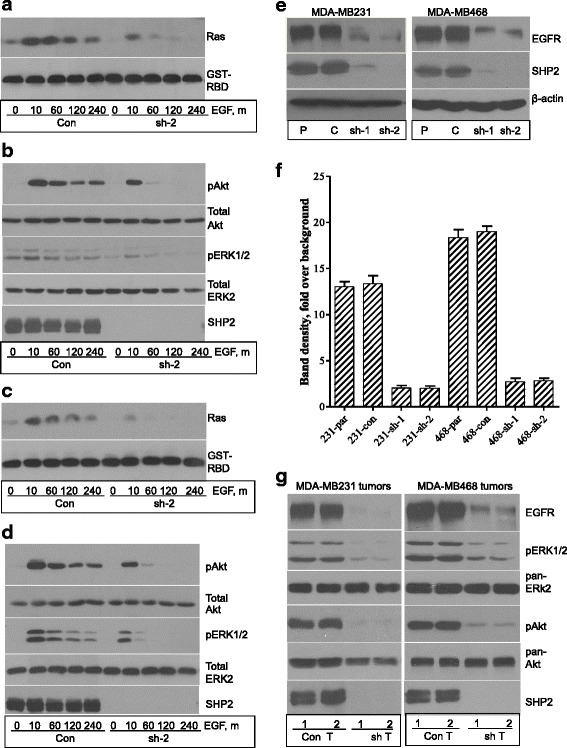
Effect of Src homology phosphotyrosyl phosphatase 2 (SHP2) silencing on epidermal growth factor (EGF)-induced signaling. Serum-starved control and SHP2-silenced cells were treated with EGF for variable times, and lysates prepared from them were analyzed for Ras, extracellular signal-regulated kinases 1 and 2 (ERK1/2), and Akt activation. **a** Analysis of Ras activation in the MDA-MB-231 cells. **b** Analysis of Akt and ERK1/2 activation in the MDA-MB-231 cells. **c** Analysis of Ras activation in the MDA-MB-468 cells. **d** Analysis of Akt and ERK1/2 activation in the MDA-MB-468 cells. **e** Analysis of epidermal growth factor receptor (EGFR) protein level in the indicated cells growing in regular (serum-containing) growth medium. **f** EGFR band density measurements done using ImageJ software (National Institutes of Health, Bethesda, MD, USA). Data presented are mean ± standard error of the mean of at least three independent experiments. In addition, the EGFR band densities in various lanes were adjusted using β-actin band densities. **g** Immunoblot analysis of tumor protein extracts for EGFR, phosphorylated ERK1/2 (pERK1/2), phosphorylated Akt (pAkt), and SHP2. *GST-RBD* glutathione *S*-transferase-fused Ras-binding domain

Dysregulation of EGFR in BTBC is suggested to promote tumorigenesis [11]. Because SHP2 is a downstream effector of EGFR signaling [14, 16, 29, 35], its positive role in BTBC could be through this mechanism. In the present study, time course EGF stimulation studies showed that EGF-induced Ras, ERK1/2, and Akt activation was suboptimal and short-lived in SHP2-silenced cells but robust and sustained in controls (Fig. 2a–d). Hence, SHP2 is essential for dysregulated EGFR signaling in BTBC cells.

Because ligand-induced receptor processing impacts signaling output, we analyzed the same total cell lysates for EGFR to see if loss of SHP2 affects this process. Surprisingly, we found that the level of EGFR in the SHP2-silenced cells was very low in the first place and rapidly degraded upon EGF stimulation (Additional file 2: Figure S2a and b). To substantiate these findings, we analyzed total cell lysates from the parental, the control, and the two SHP2 shRNA cells for basal EGFR levels and found a 6–8-fold reduction in the SHP2-silenced cells (Fig. 2e and f). We also analyzed tumor samples (Fig. 1) for EGFR protein level and ERK1/2 and Akt activation and found a drastic reduction in the SHP2-silenced tumors (Fig. 2g). To rule out the possibility of shRNA-mediated artifact, we inhibited SHP2 function by dominant-negative SHP2 expression (C459S-SHP2) and found a 5–8-fold reduction in EGFR level when compared with the wild-type counterpart or vector alone (Additional file 2: Figure S2c and d). Hence, SHP2 positively regulated EGFR expression.

### SHP2 promotes elevated EGFR expression at both protein and mRNA levels

**Fig. 3:**
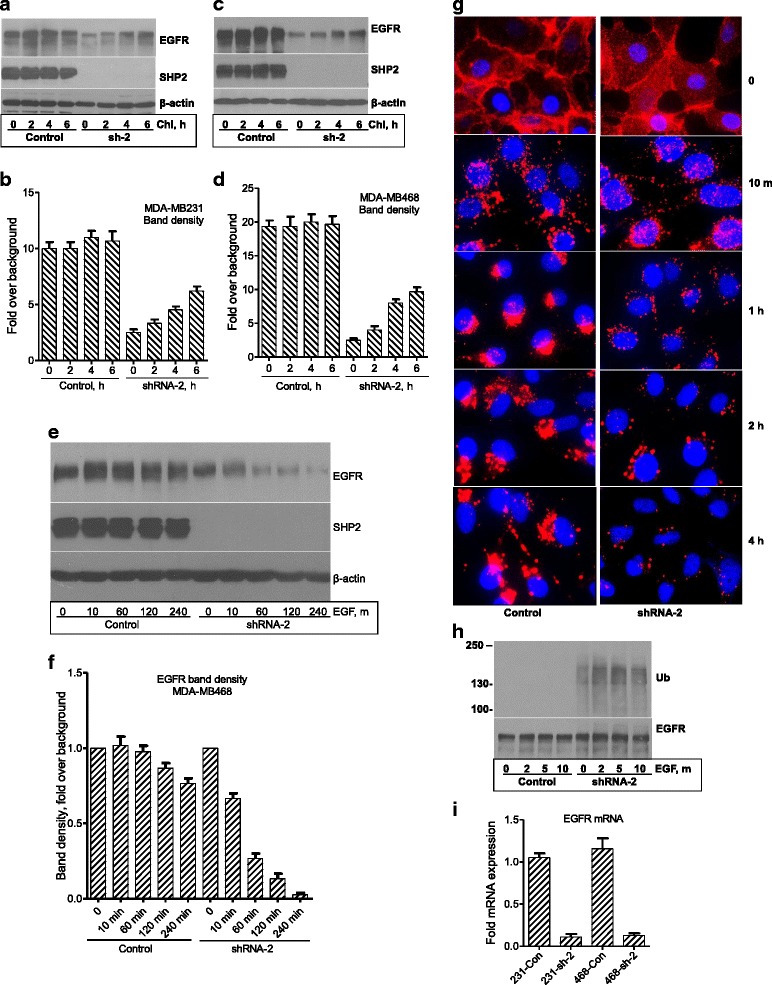
Effect of Src homology phosphotyrosyl phosphatase 2 (SHP2) silencing on epidermal growth factor receptor (EGFR) expression. Cells were treated with chloroquine (Chl) for variable times, washed, and then immediately stimulated with epidermal growth factor (EGF) for variable times. Immunoblot analysis of EGFR in the control and SHP2-silenced (sh-2) MDA-MB-231 (**a**) and MDA-MB-468 (**c**) cells treated with 100 μg/ml chloroquine for variable times. Bar graphs show EGFR band density measurements from three independent experiments in the MDA-MB-231 (**b**) and MDA-MB-468 (**d**) cells. **e** Dynamics of ligand-induced EGFR degradation in the presence and absence of SHP2. After chloroquine treatment, cells were stimulated with EGF for variable times and lysates from them were analyzed for EGFR. **f** Bar graph shows EGFR band density measurements from three independent experiments conducted as shown in (**e**). **g** Fluorescence images of control and short hairpin RNA (shRNA)-2 cells derived from the MDA-MB-468 cells. Cells were treated with tetramethylrhodamine-labeled EGF and processed as described in Materials and methods. **h** Immunoblot analysis for state of EGFR ubiquitination (Ub) in control and shRNA-2 cells after stimulation with EGF for the indicated times and immunoprecipitation with anti-EGFR antibody (*top panel*). *Bottom panel* shows comparable EGFR protein levels in all lanes. **i** Quantitative reverse transcriptase–polymerase chain reaction on EGFR mRNA levels in the control (Con) and SHP2-silenced sh-2 cells derived from the MDA-MB-231 and MDA-MB-468 cell lines. The EGFR messenger RNA (mRNA) expression level was corrected against glyceraldehyde-3-phosphate dehydrogenase mRNA in both the control and SHP2-silenced cells. The EGFR band densities in **b**, **d**, and **e** were adjusted using corresponding β-actin band densities

On the basis of the data shown in Fig. 2, we reasoned that SHP2 might act at a protein or mRNA level, or at both levels, to promote EGFR expression. Initially, we determined the effect of SHP2 on EGFR protein stability. Because membrane proteins such as EGFR are degraded primarily by the lysosomal system, we conducted lysosome inhibition studies by treating cells with 100 μg/ml chloroquine [36] for variable times. Lysosome inhibition led to a partial restoration of EGFR in the SHP2-silenced cells with minimal effect in the controls (Fig. 3a and c). Reprobing for β-actin showed comparable loading in each lane. EGFR band density measurements confirmed an approximately 50 % EGFR restoration within 6 h (Fig. 3b and d). These findings suggest that SHP2 promotes stable EGFR expression in BTBC cells at least in part by suppressing lysosomal degradation.

To corroborate the effect of SHP2 on EGFR protein stability, we studied the dynamics of ligand-induced EGFR degradation after stabilizing EGFR with chloroquine. Analysis of total cell lysates showed rapid EGFR degradation in the SHP2-silenced cells and less rapid degradation in the controls (Fig. 3e and Additional file 3: Figure S3a). Comparison of average band densities against the starting point in each group showed a 75 % EGFR drop within 1 h in the SHP2-silenced cells and only a 30 % drop within 4 h in the controls (Fig. 3f and Additional file 3: Figure S3b). These findings suggest that SHP2 suppresses EGFR degradation to promote elevated expression.

To confirm the immunoblotting findings, we conducted time course fluorescence studies after stabilizing EGFR as described above (see Materials and methods). EGF-bound EGFR was localized primarily at the plasma membrane at the zero time point. Incubation at 37 °C led to internalization within 10 min in both groups (Fig. 3g and Additional file 3: Figure S3c). Further incubation led to a rapid decay in EGF-bound EGFR in the SHP2-silenced cells, but less so in the controls. In addition, differences in receptor distribution were observed after 10 min. While EGF-bound EGFR in the controls was sorted in a polarized fashion (in reference to the nucleus), it was retained in the perinuclear region in the SHP2-silenced cells. These findings confirm the immunoblotting data and further show that SHP2 suppresses ligand-induced EGFR degradation by modulating the process of sorting.

The hypersensitivity of EGFR to ligand-induced degradation in the SHP2-silenced cells was indicative of enhanced EGFR ubiquitination. We tested this possibility after stabilizing EGFR with chloroquine and stimulating with EGF for 2, 5, or 10 min, a time range that shows maximal receptor ubiquitination. On one hand, EGFR was ubiquitinated even in the basal state in the SHP2-silenced cells, which increased upon EGF stimulation. On the other hand, EGFR ubiquitination in the controls was undetectable in the basal state and weakly detectable after EGF stimulation (Fig. 3h and Additional file 3: Figure S3d). Hence, SHP2 modulated EGFR ubiquitination to promote stability.

Because lysosome inhibition did not lead to complete restoration of EGFR, we reasoned that SHP2 might also promote EGFR expression at an mRNA level. qRT-PCR analysis showed that silencing SHP2 expression led to a 9–10-fold decrease in EGFR mRNA level (Fig. 3i). These findings show that SHP2 is important not only for EGFR protein stability but also for mRNA expression.

### SHP2 and EGFR are co-overexpressed in BTBC tumors and cell lines

On the basis of the data shown in Figs. 2 and 3, we reasoned that both EGFR and SHP2 expression might be dysregulated in BTBC due to stoichiometric requirements. We thus analyzed a total of 56 BTBC tumors for EGFR and SHP2 co-overexpression by IHC and IF. In order to include surrounding normal tissue as an internal control, the classical approach of sectioning the whole resected tissue samples was employed.

**Fig. 4:**
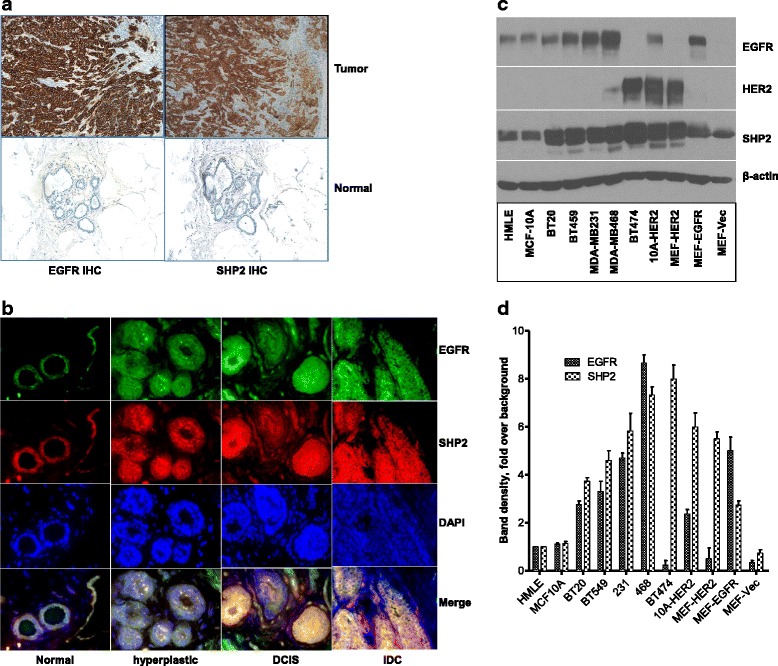
Analysis of epidermal growth factor receptor (EGFR) and Src homology phosphotyrosyl phosphatase 2 (SHP2) expression in basal-like and triple-negative breast cancer (BTBC) tumors and cell lines. **a** Representative 3+ immunohistochemistry (IHC) pictures of SHP2 and EGFR expression in tumor and adjacent normal tissue. **b** Immunofluorescence costaining for SHP2 and EGFR showing state of expression in normal-looking, hyperplastic, ductal carcinoma in situ (DCIS), and infiltrating ductal carcinoma (IDC) regions of a representative specimen. Note that the level of expression for both proteins is comparable in various stages of tumor development. **c** Analysis of SHP2 and EGFR co-overexpression in breast cancer cell lines and in experimentally produced EGFR-overexpressing cells. The BT-474 breast cancer cell line that overexpresses human epidermal growth factor receptor 2 (HER2), MCF-10A, and mouse embryonic fibroblasts (MEFs) ectopically overexpressing HER2 and/or EGFR was also used in this analysis. Parental human mammary luminal epithelial cells (HMLE), MCF-10A cells, and MEFs were used as negative controls for receptor tyrosine kinase overexpression. **d** EGFR and SHP2 band density measurements from at least three independent experiments analyzed as shown in (**c**). The EGFR (*open bar*) and SHP2 (*hatched bar*) band densities in the various lanes were adjusted using β-actin band densities. *DAPI* 4′,6-diamidino-2-phenylindole

Scores of 3+ and 2+ for each protein were considered as positive for elevated expression. Representative pictures of EGFR and SHP2 expression in malignant and surrounding normal tissue from two cases are shown in Fig. 4a and Additional file 4: Figure S4a. Of the 56 tumors, 39 (approximately 70 %) and 33 (approximately 59 %) were positive for elevated SHP2 and EGFR expression, respectively (Table 1 and Additional file 6: Table S1). Comparison of EGFR and SHP2 IHC scores in each tumor showed a tight association in expression. We found that 31 (94 %) of the 33 EGFR-positive tumors were also positive for SHP2 (Additional file 6: Table S1).

**Table 1:** Analysis of 56 basal-like and triple-negative breast cancer tumor samples

Proteins analyzed	IHC score, *n*				Overexpressed, *n* (%)
	3+	2+	1+	0	
SHP2	22	17	11	6	39 (70 %)
EGFR	13	20	9	5	33 (59 %)

*EGFR* epidermal growth factor receptor, *IHC* immunohistochemistry, *SHP2* Src homology phosphotyrosyl phosphatase 2

A total of 56 basal-like and triple-negative breast cancer tumor samples were analyzed for SHP2 and EGFR by immunohistochemistry as described in Materials and methods. Samples that showed 3+ and 2+ expression levels were considered as positive for elevated expression of SHP2 and EGFR

Tumors positive for both proteins were further analyzed by IF microscopy to confirm co-overexpression. In these analyses, we also paid particular attention to heterogeneous regions in each tumor. Compared with the normal-looking regions, overexpression of EGFR and SHP2 increased with tumor progression. Representative IF pictures from two cases are shown in Fig. 4b and Additional file 4: Figure S4b. These results confirm the IHC results and further suggest that SHP2 and EGFR are co-overexpressed in all stages of BTBC development, with the level of expression increasing with disease progression.

We also determined the state of EGFR and SHP2 expression in multiple BTBC cell lines (BT-20, BT-549, MDA-MB-231, and MDA-MB-468) to confirm the IHC and IF findings. The HER2-positive BT-474 breast cancer cell line overexpressing SHP2 [19] and the MCF-10A cells and MEFs ectopically overexpressing HER2 and EGFR were used as positive controls, while parental MCF-10A cells and MEFs as well as HMLE cells were used as negative controls for overexpression. Consistent with the findings in BTBC tumors, EGFR and SHP2 were co-overexpressed in BTBC cells (Fig. 4c). Compared with parental MCF-10A or HMLE cells, EGFR and SHP2 were overexpressed 4–6-fold in BTBC and experimentally produced cells (Fig. 4d). Also, the positive control BT-474 cells had more than 7-fold SHP2. Hence, co-overexpression of SHP2 and EGFR is commonplace.

### SHP2 inhibition is superior to EGFR inhibition in suppressing cell transformation

**Fig. 5:**
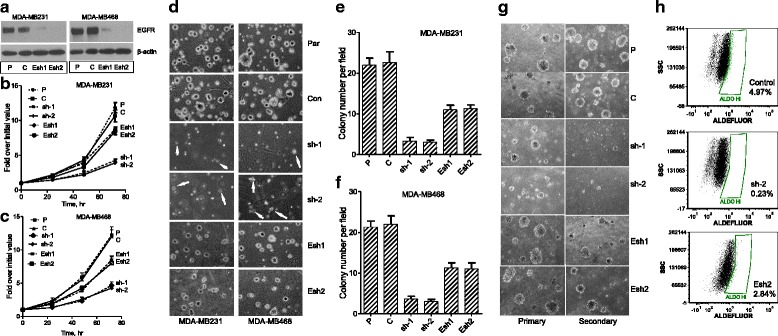
Effect of Src homology phosphotyrosyl phosphatase 2 (SHP2) and epidermal growth factor receptor (EGFR) silencing on cell proliferation, anchorage-independent growth, and mammosphere formation. **a** Immunoblotting analysis of EGFR silencing with two independent short hairpin RNA (shRNA) constructs in the MDA-MB-231 and MDA-MB-468 cells. **b** Effect of EGFR and SHP2 silencing on cell proliferation in MDA-MB-231 cells. **c** Effect of EGFR and SHP2 silencing on cell proliferation in MDA-MB-468 cells. **d** Effect of EGFR and SHP2 silencing on anchorage-independent growth in soft agar. **e** Colony numbers from three independent experiments in MDA-MB-231 cells cultured as shown in (**d**). **f** Colony numbers from three independent experiments in MDA-MB-468 cells cultured as shown in (**d**). **g** Effect of EGFR and SHP2 silencing on mammosphere formation in MDA-MB-231 cells. **h** Effect of EGFR and SHP2 silencing on proportion of aldehyde dehydrogenase 1 (ALDO)-high cells in MDA-MB-231 cells. *P* parental, *C* control, *sh-1* SHP2 shRNA-1, *sh-2* SHP2 shRNA-2, *Esh1* EGFR shRNA-1, *Esh2* EGFR shRNA-2, *SSC* side scatter

Because SHP2 is important for EGFR expression and signaling, we reasoned that its positive role in BTBC might be through this mechanism. To test this possibility, we compared the relative biological significance of SHP2 and EGFR in BTBC cells. First, the expression of EGFR was silenced using two shRNA constructs (Fig. 5a) that were previously shown to be specific [26]. Next, we compared the effect of SHP2 and EGFR silencing on cell proliferation. While SHP2 inhibition suppressed cell proliferation by about 3–4-fold, EGFR inhibition led to only 1.5-fold suppression of cell proliferation within 3 days (Fig. 5b and c). Thus, SHP2 inhibition is more efficient than EGFR inhibition in suppressing cell growth.

We also compared the effect of SHP2 and EGFR silencing on anchorage-independent growth in soft agar. On one hand while the parental and the control cells formed larger and more numerous colonies, the SHP2-silenced cells formed smaller and fewer ones (Fig. 5d). On the other hand, silencing EGFR led to a modest decrease in colony number only. Viewing under the 4× lens objective showed 22 larger colonies per field in the controls and only 2–4 smaller colonies in the SHP2-silenced cells (Fig. 5e and f). With regard to EGFR, the average colony number in silenced cells was approximately 11 per field. These results suggest that SHP2 plays a major role in cell transformation, while EGFR makes a significant but relatively modest contribution.

The inability of the SHP2-silenced cells to grow in soft agar indicated a role for SHP2 in cancer stem cell (CSC) biology. To verify this point, we compared the mammosphere-forming capacity of SHP2- and EGFR-silenced cells in suspension cultures in which cells with stem-like properties only can survive and form these structures [37, 38]. On one hand, whereas mammosphere-forming efficiency in the parental and control cells was increased on successive passaging from primary to secondary cultures, it was exhausted in the SHP2-silenced cells (Fig. 5g and Additional file 5: Figure S5a). On the other hand, the impact of EGFR silencing was noticeable only in secondary cultures, where a modest decrease in mammosphere number was observed. A further property of CSC is increased ALDH1 activity [39] as determined using the ALDEFLUOR assay. We used this assay to confirm the mammosphere findings. On one while the control MDA-MB-231 cells had 4.97 % ALDH1-high cells, the corresponding SHP2-silenced cells had only 0.23 %, a more than 20-fold reduction (Fig. 5h). On the other hand, the EGFR-silenced cells had 2.3 % ALDH1-high cells, suggesting a relatively modest decrease. Similar results were obtained with the MDA-MB-468 cells (Additional file 5: Figure S5b). Thus, SHP2 is more important than EGFR in promoting CSC phenotypes in BTBC cells.

### SHP2 promotes the expression and signaling of FGFR1 and c-Met in BTBC cells

**Fig. 6:**
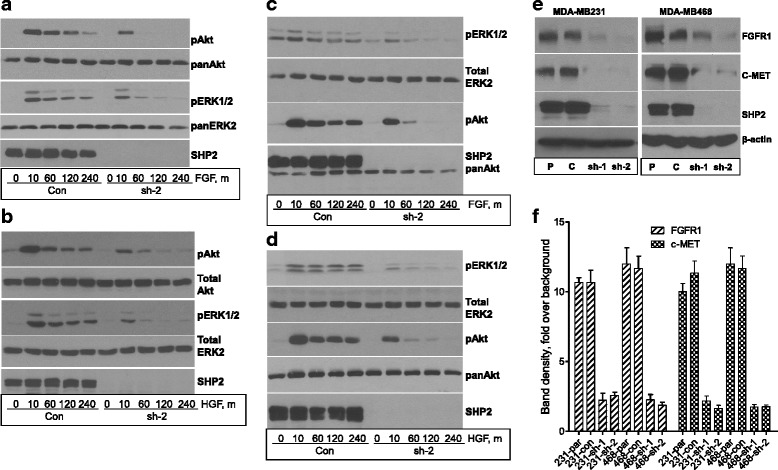
Effect of Src homology phosphotyrosyl phosphatase 2 (SHP2) silencing on fibroblast growth factor receptor (FGFR) and c-Met expression and signaling. **a** Effect of SHP2 silencing on fibroblast growth factor (FGF)-induced signaling in the MDA-MB-231 cells. **b** Effect of SHP2 silencing on FGF-induced signaling in the MDA-MB-468 cells. **c** Effect of SHP2 silencing on hepatocyte growth factor (HGF)-induced signaling in the MDA-MB-231 cells. **d** Effect of SHP2 silencing on HGF-induced signaling in the MDA-MB-468 cells. Note that silencing SHP2 expression led to suboptimal and short-lived FGF- or HGF-induced extracellular signal-regulated kinases 1 and 2 (ERK1/2) and Akt activation in both cell lines. **e** Effect of SHP2 silencing on the protein levels of FGFR1 and c-Met in the MDA-MB-231 and MDA-MB-468 cells. **f** Average of band density measurements for FGFR and c-MET from three independent experiments. *Con* controls, *sh-1* SHP2 short hairpin RNA-1, *sh-2* SHP2 short hairpin RNA-2

The prominence of SHP2 inhibition over that of EGFR in suppressing cell transformation was suggestive of SHP2 controlling additional RTKs that are known to be dysregulated in BTBC, such as c-Met and FGFR1 [3–5]. We thus determined the effect of SHP2 silencing on FGFR and c-Met signaling in the same BTBC cells. Serum-starved cells were stimulated with either FGF or hepatocyte growth factor (HGF) for varying times, and lysates prepared from them were analyzed for ERK1/2 and Akt activation. Both MDA-MB-231 and MDA-MB-468 cells were highly responsive to stimulation with either ligand as determined by a robust activation of ERK1/2 and Akt. While HGF- and FGF-induced ERK1/2 and Akt activation was suboptimal and short-lived in the SHP2-silenced cells, it was augmented and sustained in the controls (Fig. 6a–d). Hence, SHP2 regulates signaling of multiple RTKs in BTBC cells.

As described in Fig. 2, SHP2 promotes EGFR expression. We thus reasoned that SHP2 might also promote c-Met and FGFR1 expression in BTBC cells. To address this point, we analyzed the protein levels of both receptors under basal growth conditions by immunoblotting total cell lysates with specific antibodies. Similarly to EGFR, the protein levels of FGFR1 and c-Met were very low in the SHP2-silenced cells (Fig. 6e). Band density measurements confirmed that the levels of FGFR1 and c-Met in the SHP2-silnced cells were lower by 4–5-fold compared with the parental and the control cells (Fig. 6f). Hence, SHP2 is essential for the expression of FGFR and c-Met in BTBC cells, and its inhibition leads to their downregulation.

## Discussion

Dysregulation of multiple RTKs, including EGFR, FGFR1, and HGFR (also called *c-Met*) [3–5], is one of the major molecular aberrations in BTBC. As a result, efforts have been made to develop RTK-targeting therapies to treat BTBC. However, the results so far show that anti-RTK drugs are ineffective when given individually and toxic when administered in combination [3, 40]. It is therefore imperative to discover and target master regulators of RTK signaling to overcome this obstacle. In this report, we have presented evidence showing that SHP2 functions as a master regulator of RTK expression and signaling in BTBC, suggesting its potential for targeted therapy in BTBC.

Recently, we demonstrated that SHP2 promotes the transformation and invasive properties of BTBC cells [24]. However, its role in tumorigenesis in vivo was not determined. In this report, we have demonstrated that inhibition of SHP2 effectively suppresses tumor growth induced by intramammary transplantation of MDA-MB-231 and MDA-MB-468 cells (Fig. 1b and Additional file 1: Figure S1a). One of the most interesting observations in mice bearing the control MDA-MB-231 tumors was the development of clinical symptoms such as increased breathing rate and reduced demeanor while the tumors were still small. Silencing SHP2 expression effectively blocked the development of these symptoms. Histopathological analysis later showed extensive lung and liver metastatic lesions in the control mice but not in the SHP2 shRNA mice (Fig. 1d and e), validating the observed clinical symptoms. These findings mimic the pathogenesis of BTBC in women in whom metastasis is often observed while the tumors are still small. Hence, inhibition of SHP2 in BTBC may mitigate distant metastasis.

Another interesting observation was that mice bearing SHP2-silenced MDA-MB-231 tumors survived far beyond the control group without manifesting any clinical symptoms (Fig. 1c). Similar results were obtained with the MDA-MB-468 cells in terms of tumor burden, which is the major clinical manifestation with this cell line (Additional file 1: Figure S1b). These findings imply that inhibition of SHP2 has the potential to provide a survival benefit to BTBC patients.

Although the positive role of SHP2 in normal RTK signaling is known [14–17], its role in dysregulated RTK signaling in cancer is unclear. In this report, we have shown that EGFR, the most commonly dysregulated RTK in BTBC [41–43], cannot effectively activate downstream signaling without SHP2 (Fig. 2a–d). Unexpectedly, we discovered a new role for SHP2: promoting elevated EGFR expression in BTBC cells (Fig. 2e and f and Additional file 2: Figure S2a–d). Hence, SHP2 functions not only downstream but also upstream of the EGFR.

One of the mechanisms for SHP2 in promoting elevated EGFR expression was through blocking ubiquitination and ligand-induced degradation (Fig. 3 and Additional file 3: Figure S3). However, our findings are in contrast to those in a recent report that suggested otherwise on the basis of transient expression of dominant-negative SHP2 with c-Cbl and Spry2 in COS1 cells [44]. In our studies, expression of dominant-negative SHP2 downregulates EGFR (Additional file 2: Figure S2c). These discrepancies might be related to cellular context and technical differences used in the two studies. While in our studies we used constitutive SHP2 inhibition with two different approaches (shRNA and dominant-negative SHP2), the researchers in the other study used transient co-overexpression. Nonetheless, our data are consistent with SHP2 positively regulating EGFR protein stability.

The incomplete recovery of EGFR by lysosome inhibition was an indication of the existence of additional mechanisms used by SHP2 to promote elevated EGFR expression. Indeed, the qRT-PCR analysis showed an approximately 16-fold reduction in EGFR mRNA level in SHP2-silenced BTBC cells (Fig. 2i). These findings add more complexity to the mechanism of SHP2 in regulating EGFR expression. They suggest that SHP2 promotes elevated EGFR expression at both protein and mRNA levels.

The tight association of EGFR and SHP2 overexpression in BTBC tumors (Fig. 4, Additional file 4: Figure S4 and Table 1) and the positive role of SHP2 in EGFR expression in BTBC cells (Figs. 2 and 3) suggest that SHP2 might also promote EGFR overexpression in BTBC patient tumors. Although our sample size was relatively small, the SHP2 and EGFR results are in agreement with previously reported data [11, 18, 19, 45, 46], supporting the validity of our data. However, the mechanism that leads to elevated SHP2 expression and promotion of EGFR mRNA level by SHP2 are unclear at this stage. Future studies addressing these questions might be needed.

We have demonstrated that SHP2 plays a major role, while EGFR makes a modest contribution, in promoting BTBC cell proliferation, anchorage-independent growth, and CSC phenotypes (Fig. 5 and Additional file 5: Figure S5). The complementarity of the mammosphere and ALDH1 findings suggests that SHP2 plays a pivotal role in CSC survival and propagation, minority cell populations known to perpetuate tumor growth, metastasis, and drug resistance [37, 38]. Thus, it is entirely possible that inhibition SHP2 might lead to elimination of CSCs in tumors, but future studies addressing this point are needed.

The superiority of SHP2 inhibition over EGFR inhibition led to the discovery that SHP2 also controls the expression and signaling activities of FGFR1 and c-Met (Fig. 6), two other RTKs known to be dysregulated in BTBC. The most novel finding was that SHP2 is essential not only for downstream signaling but also for expression of both FGFR1 and c-Met. Although our data do not delineate between gene expression and protein stability, we speculate that SHP2 promotes FGFR1 and c-Met expression by acting at both protein and mRNA levels, similar to its role in EGFR signaling. Future studies are needed to verify these points.

## Conclusions

In this report, we show that inhibition of SHP2 blocks BTBC tumorigenesis and/or metastasis. We also show that SHP2 mediates the expression and signaling activities of multiple RTKs, including EGFR, FGFR1, and c-Met, to promote BTBC. To our knowledge, this is the first report to show that SHP2 acts both upstream and downstream of RTKs to promote their oncogenic signaling. This effect of SHP2 might be responsible for its superior role compared with EGFR inhibition. Subsequent studies are needed to answer some of the questions raised in this report.

## Additional files

Additional file 1: Figure S1. Effect of SHP2 silencing on the tumorigenic potential of MDA-MB-468 cells. a Tumor growth rate as determined by tumor volume measurement. b Survival plot based on tumor size. Mice that bore tumor volume of approximately 2000 mm^3^ or more were considered as not surviving and those with tumor volume below 750 mm^3^ as surviving during the study period. For determining survival, data from six control and six SH-2 mice was used. c H & E staining of mammary tumor sections. Note that the control tumor shows local invasiveness, while the shRNA tumor does not show any obvious pushing front and invasiveness. d H & E staining of lung and liver sections harvested from the control and shRNA mice that received transplanted MDA-MB-468 cells. (PDF 695 kb) Additional file 2: Figure S2. Silencing SHP2 expression in the MDA-MB-231 (a) and MDA-MB-468 (b) cells drastically reduced EGFR protein level, which became hypersensitive to EGF-induced degradation. Inhibition of SHP2 by dominant-negative (C459S-SHP2) expression led to a similar decrease (similar to shRNA-based inhibition) in EGFR protein level, but expression of vector alone or wild-type SHP2 (WT-SHP) did not. (a) Immunoblotting data and (b) bar graph showing band density measurement values of the immunoblotting data. The values represent data from at least three independent experiments. (PDF 201 kb) Additional file 3: Figure S3. Dynamics of EGFR degradation in the MDA-MB-231 cells in the presence and absence of SHP2. a First EGFR was stabilized by chloroquine treatment for 6 h, and then cells were stimulated with 10 ng/ml EGF for the indicated times. Lysates prepared from these cells were analyzed by immunoblotting with anti-EGFR antibody. As shown, EGFR was degraded relatively rapidly in the SHP2-silenced cells but not in the controls. The anti-SHP2 and anti-β-actin immunoblotting show efficient SHP2 silencing and comparable total protein loading, respectively. b Band density measurement of the EGFR immunoblotting. As shown, approximately 75 % of EGFR in the SHP2-silnced cells was degraded within 1 h, but only 30 % was degraded in the controls even at the 4-h time point. Data shown are mean ± standard error of three independent experiments. With regard to the effect of SHP2 silencing on EGFR endocytosis and processing in the MDA-MB-231 cells, EGF-induced EGFR endocytosis was affected not by SHP2 silencing, but by the processing thereafter. While EGFR in the controls was sorted in polarized fashion with gradual outward extension, it remained perinuclear in the SHP2 silenced cells. In addition, it was possible to discern the rapid dissipation of the EGFR signal in the SHP2-silenced cells but not in the controls. With regard to the effect of SHP2 silencing on EGFR ubiquitination in the MDA-MB-231 cells, first EGFR was stabilized by chloroquine treatment for 6 h and then cells were stimulated with 10 ng/ml EGF for the indicated times. Lysates prepared from these cells were subjected to immunoprecipitation with anti-EGFR and immunoblotting with anti-ubiquitin antibodies. To compensate for the partial EGFR restoration by chloroquine, the amount of EGFR precipitate loaded in the shRNA lanes was increased by 25 %. As shown, EGFR was rapidly and highly ubiquitinated in the SHP2-silenced cells but less so in the controls. Reprobing with anti-EGFR antibody showed comparable protein levels in all lanes. (PDF 310 kb) Additional file 4: Figure S4. EGFR and SHP2 IHC staining of a BTBC tumor. As shown, the tumor (Tum) is 3+ for both SHP2 and EGFR, while the corresponding normal (Nor) tissue is negative for both proteins. Immunofluorescent staining of a BTBC tumor section shows the expression of EGFR and SHP2, which was analyzed by costaining. As shown, the IF intensity was low in the normal region, medium in the hyperplastic region, and higher in the ductal carcinoma in situ (DCIS) and infiltrating ductal carcinoma (IDC) regions. (PDF 437 kb) Additional file 5: Figure S5. a Effect of SHP2 and EGFR silencing on primary and secondary mammosphere formation by the MDA-MB-468 cells. Note that the mammosphere-forming capacity of the SHP2-silenced cells was exhausted in secondary passaging, while a modest reduction was observed in the EGFR-silenced cells. b Effect of SHP2 and EGFR silencing on proportion of ALDH1-high cells. Silencing SHP2 expression drastically reduced the ALDH1-high cells, but silencing EGFR had only a modest effect. (PDF 237 kb) Additional file 6: Table S1. Internal codes for each case and the state of EGFR and SHP2 expression for each tumor. The first 12 cases were reanalyses of samples from a previous publication [14] in which the indicated samples were reported as triple-negative. Cases 84–127 are new BTBC samples. Yellow highlighting represents the co-overexpression of SHP2 and EGFR, *cyan blue* represents SHP2-positive tumors that do not have EGFR overexpression, and *gray* represents EGFR-positive tumors without SHP2 overexpression. (DOC 66 kb)

## Abbreviations

ALDH1: aldehyde dehydrogenase 1
BTBC: basal-like and triple-negative breast cancer
Chl: chloroquine
CSC: cancer stem cell
DAPI: 4′,6-diamidino-2-phenylindole
DCIS: ductal carcinoma in situ
EGF: epidermal growth factor
EGFR: epidermal growth factor receptor
ERK1/2: extracellular signal-regulated kinases 1 and 2
FGF: fibroblast growth factor
FGFR: fibroblast growth factor receptor
GAPDH: glyceraldehyde-3-phosphate dehydrogenase
GST-RBD: glutathione *S*-transferase-fused Ras-binding domain
H & E: hematoxylin and eosin
HER2: human epidermal growth factor receptor 2
HGF: hepatocyte growth factor
HGFR: hepatocyte growth factor receptor
HMLE: human mammary luminal epithelial cells
IDC: infiltrating ductal carcinoma
IF: immunofluorescence
IHC: immunohistochemistry
MEF: mouse embryonic fibroblast
NOD/SCID: non-obese diabetic/severe combined immunodeficiency
qRT-PCR: quantitative reverse transcriptase–polymerase chain reaction
RTK: receptor tyrosine kinase
SH2: Src homology 2
SHP2: Src homology phosphotyrosyl phosphatase 2
shRNA: short hairpin RNA
