# Supplementary material for: SHP2 acts both upstream and downstream of multiple receptor tyrosine kinases to promote basal-like and triple-negative breast cancer
Source: Breast Cancer Res. 2016 Jan 4;18:2. doi: 10.1186/s13058-015-0659-z (PMC4700603; doi:10.1186/s13058-015-0659-z)
Supplement: Additional file 1: Figure S1. — Effect of SHP2 silencing on the tumorigenic potential of MDA-MB-468 cells. a Tumor growth rate as determined by tumor volume measurement. b Survival plot based on tumor size. Mice that bore tumor volume of approximately 2000 mm3 or more were considered as not surviving and those with tumor volume below 750 mm3 as surviving during the study period. For determining survival, data from six control and six SH-2 mice was used. c H & E staining of mammary tumor sections. Note that the control tumor shows local invasiveness, while the shRNA tumor does not show any obvious pushing front and invasiveness. d H & E staining of lung and liver sections harvested from the control and shRNA mice that received transplanted MDA-MB-468 cells. (PDF 695 kb) [file 13058_2015_659_MOESM1_ESM.pdf]

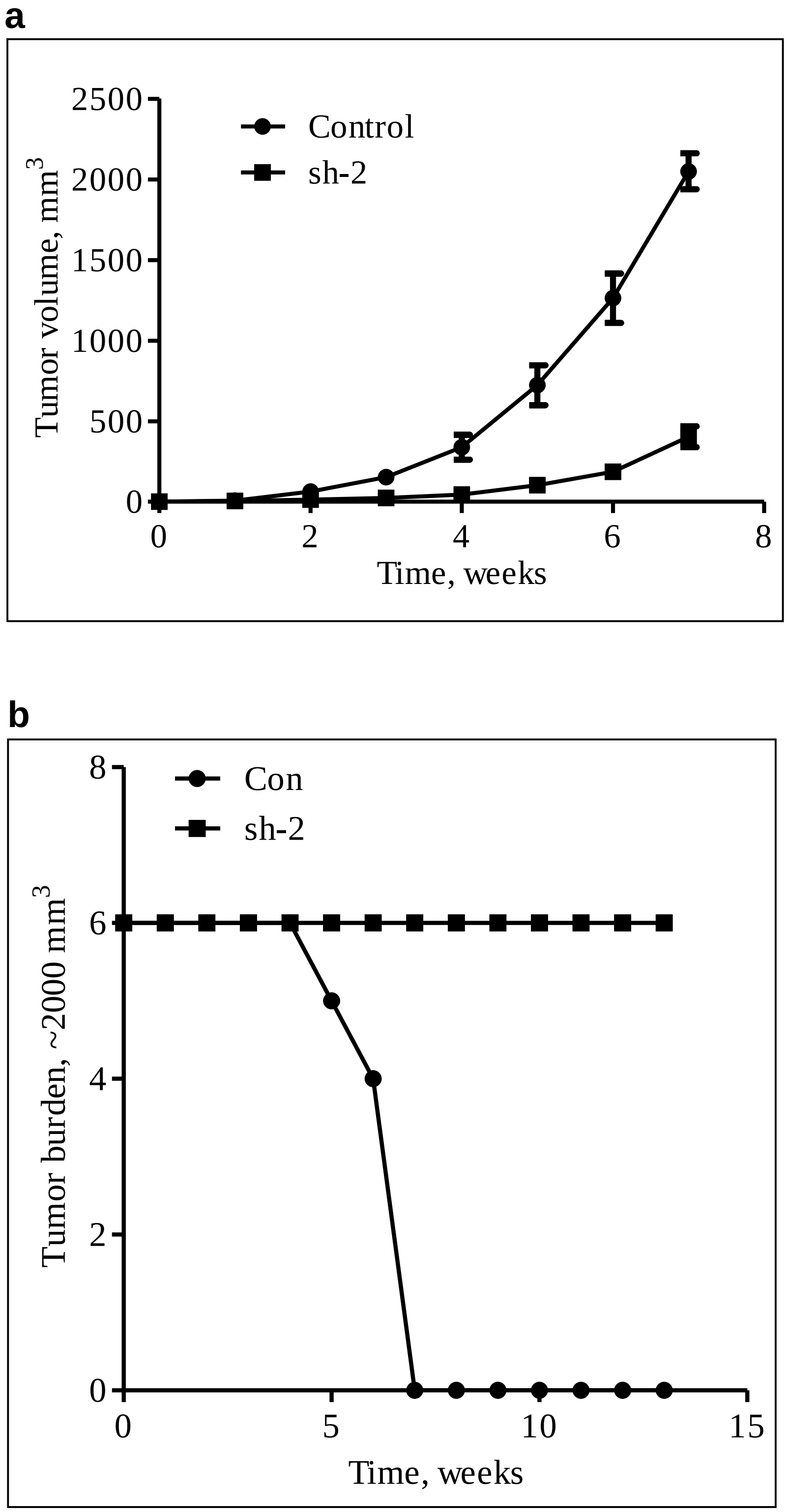

Supplementary figure 1: Effect of SHP2 silencing on the tumorigenic potential of MDA-MB468 cells. **a**) Tumor growth rate as determined by tumor volume measurement. **b**) Survival plot based on tumor size. Mice that bore tumor volume of approximately 2000 mm^3 or more were considered as not surviving, while those showing sizes below 750 mm^3 as surviving during the study period. For determining survival, data from 6 control and 6 sh-2 mice was used.

c

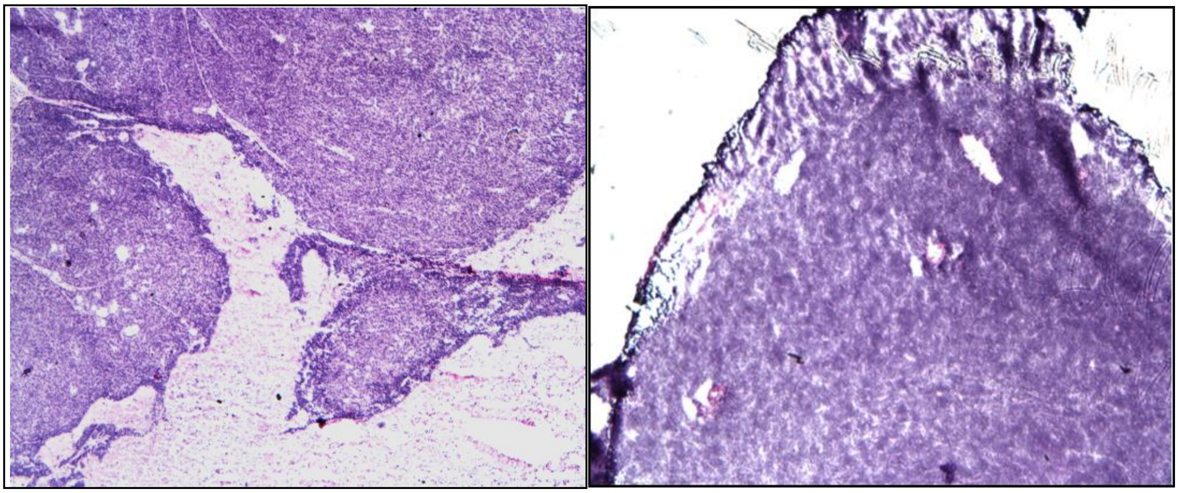

Control shRNA-2

d

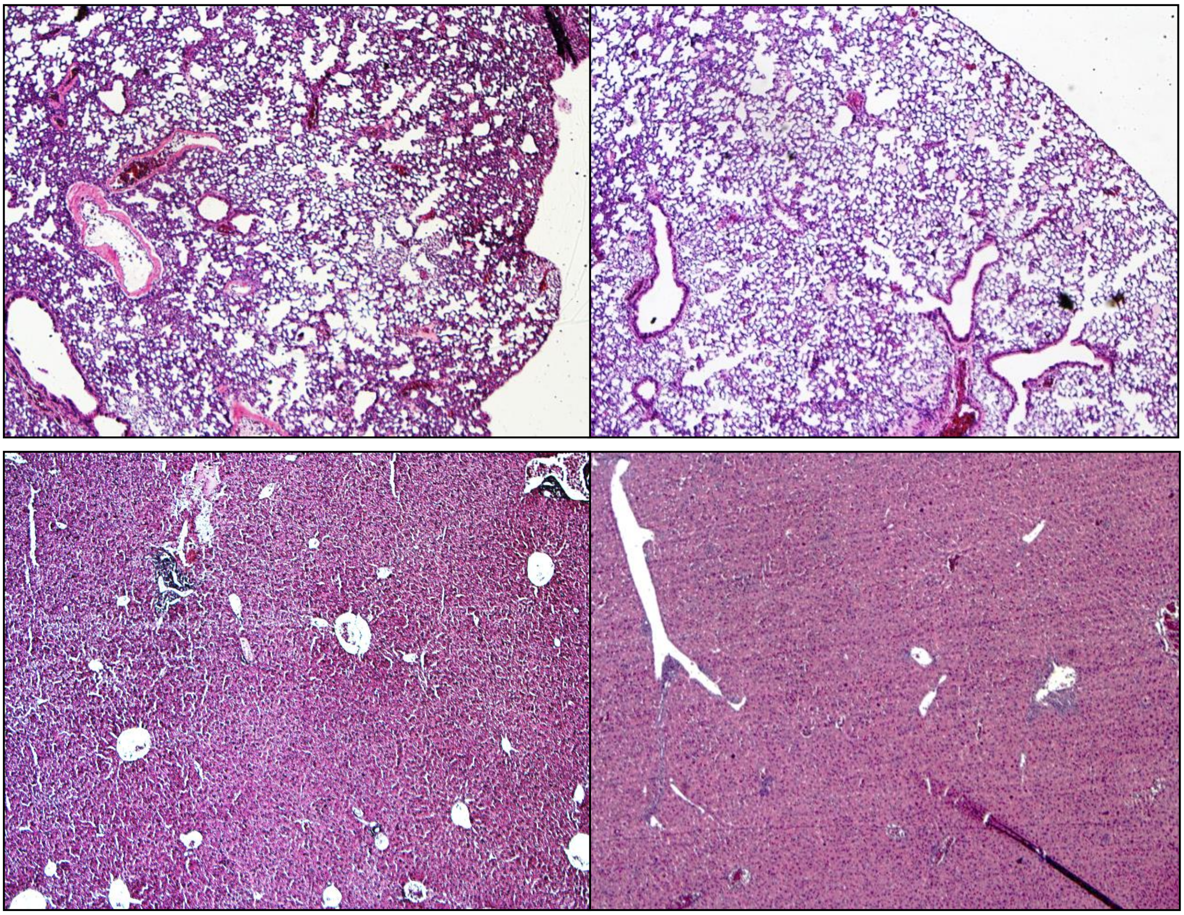

Lung

Liver

Control shRNA-2

Supplementary figure 1: C) H & E staining of mammary tumor sections. Note that the control tumor shows local invasiveness while the shRNA tumor doers not show any obvious pushing front and invasiveness. **D**) H & E staining of lung and liver sections harvested from the control and shRNA mice transplanted with MDA-MB468 cells.
