# Supplementary material for: SHP2 acts both upstream and downstream of multiple receptor tyrosine kinases to promote basal-like and triple-negative breast cancer
Source: Breast Cancer Res. 2016 Jan 4;18:2. doi: 10.1186/s13058-015-0659-z (PMC4700603; doi:10.1186/s13058-015-0659-z)
Supplement: Additional file 2: Figure S2. — Silencing SHP2 expression in the MDA-MB-231 (a) and MDA-MB-468 (b) cells drastically reduced EGFR protein level, which became hypersensitive to EGF-induced degradation. Inhibition of SHP2 by dominant-negative (C459S-SHP2) expression led to a similar decrease (similar to shRNA-based inhibition) in EGFR protein level, but expression of vector alone or wild-type SHP2 (WT-SHP) did not. (a) Immunoblotting data and (b) bar graph showing band density measurement values of the immunoblotting data. The values represent data from at least three independent experiments. (PDF 201 kb) [file 13058_2015_659_MOESM2_ESM.pdf]

A

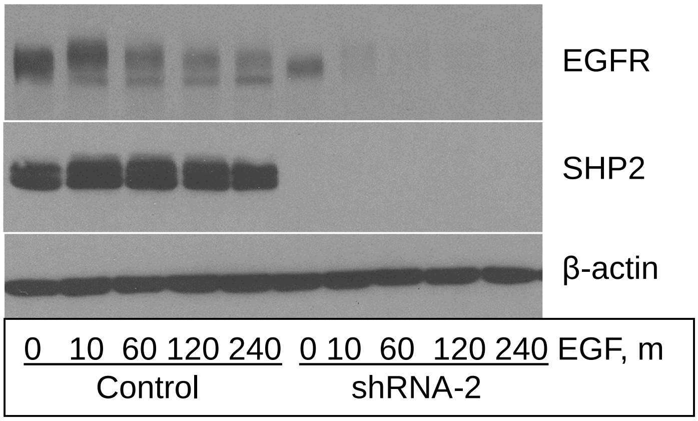

B

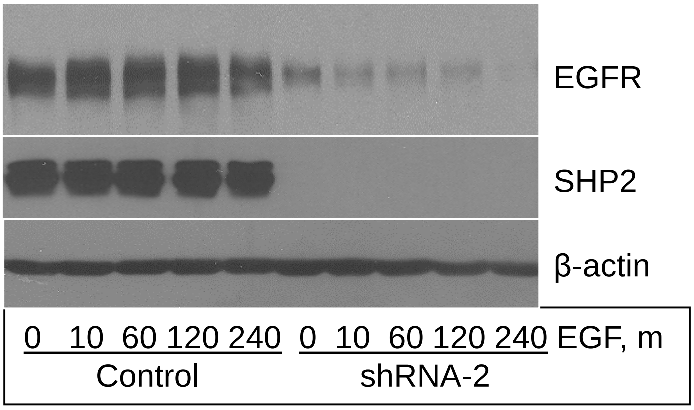

Supplementary Figure 2A and B: Silencing SHP2 expression in the MDA-MB231 (**A**) and MDA-MB468 (**B**) cells drastically reduced EGFR protein level, which became hypersensitive to EGF-induced degradation.

C

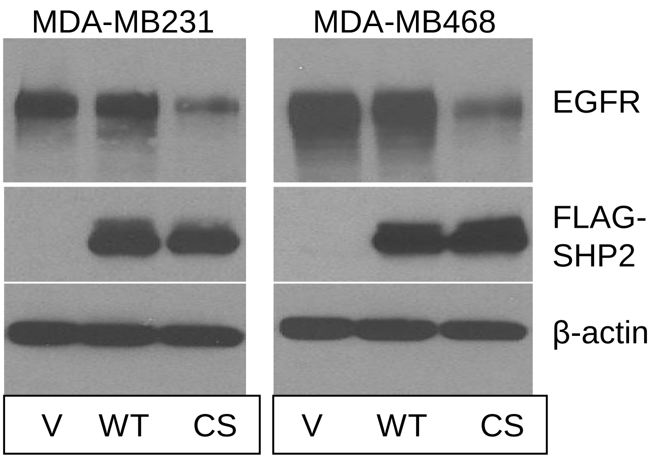

D

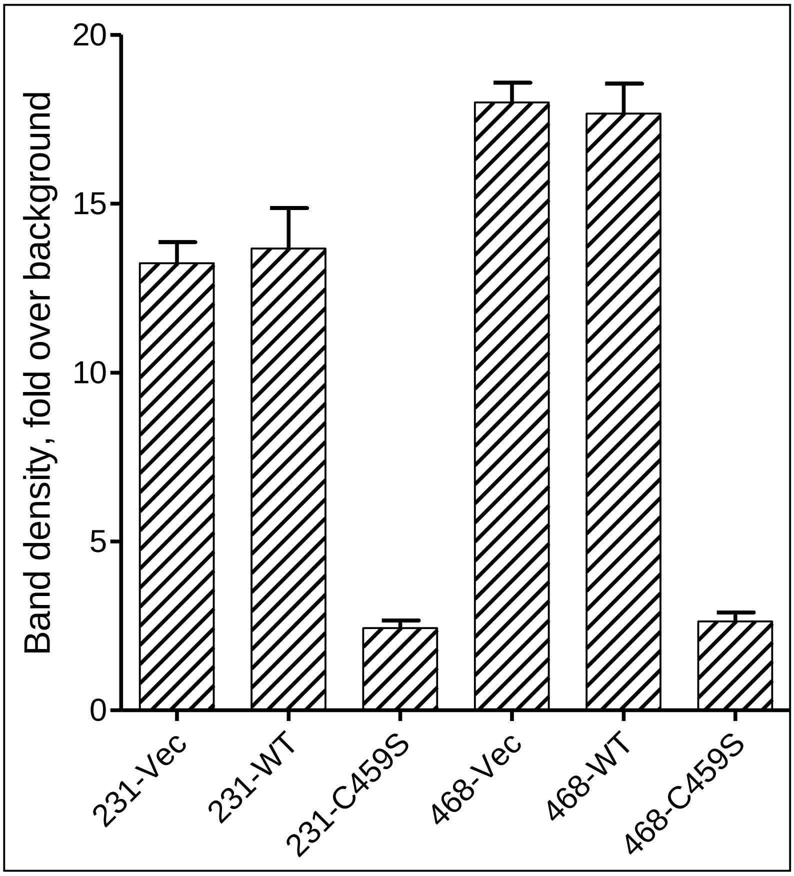

Supplementary Figure 2C and D: Inhibition of SHP2 by dominant-negative (C459S-SHP2) expression led to a similar decrease (similar to shRNA-based inhibition) in EGFR protein level, but expression of vector alone or wild-type SHP2 (WT-SHP) did not. **A**) Immunoblotting data and **B**) bar graph showing band density measurement values of the immunoblotting data. The values represent at least three independent experiments.
