# Supplementary material for: SHP2 acts both upstream and downstream of multiple receptor tyrosine kinases to promote basal-like and triple-negative breast cancer
Source: Breast Cancer Res. 2016 Jan 4;18:2. doi: 10.1186/s13058-015-0659-z (PMC4700603; doi:10.1186/s13058-015-0659-z)
Supplement: Additional file 3: Figure S3. — Dynamics of EGFR degradation in the MDA-MB-231 cells in the presence and absence of SHP2. a First EGFR was stabilized by chloroquine treatment for 6 h, and then cells were stimulated with 10 ng/ml EGF for the indicated times. Lysates prepared from these cells were analyzed by immunoblotting with anti-EGFR antibody. As shown, EGFR was degraded relatively rapidly in the SHP2-silenced cells but not in the controls. The anti-SHP2 and anti-β-actin immunoblotting show efficient SHP2 silencing and comparable total protein loading, respectively. b Band density measurement of the EGFR immunoblotting. As shown, approximately 75 % of EGFR in the SHP2-silnced cells was degraded within 1 h, but only 30 % was degraded in the controls even at the 4-h time point. Data shown are mean ± standard error of three independent experiments. With regard to the effect of SHP2 silencing on EGFR endocytosis and processing in the MDA-MB-231 cells, EGF-induced EGFR endocytosis was affected not by SHP2 silencing, but by the processing thereafter. While EGFR in the controls was sorted in polarized fashion with gradual outward extension, it remained perinuclear in the SHP2 silenced cells. In addition, it was possible to discern the rapid dissipation of the EGFR signal in the SHP2-silenced cells but not in the controls. With regard to the effect of SHP2 silencing on EGFR ubiquitination in the MDA-MB-231 cells, first EGFR was stabilized by chloroquine treatment for 6 h and then cells were stimulated with 10 ng/ml EGF for the indicated times. Lysates prepared from these cells were subjected to immunoprecipitation with anti-EGFR and immunoblotting with anti-ubiquitin antibodies. To compensate for the partial EGFR restoration by chloroquine, the amount of EGFR precipitate loaded in the shRNA lanes was increased by 25 %. As shown, EGFR was rapidly and highly ubiquitinated in the SHP2-silenced cells but less so in the controls. Reprobing with anti-EGFR antibody showed comparable protein levels in all lane [file 13058_2015_659_MOESM3_ESM.pdf]

a

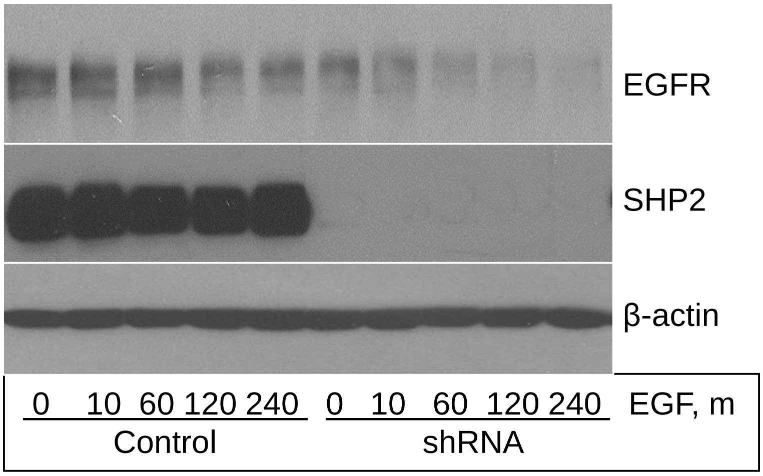

B

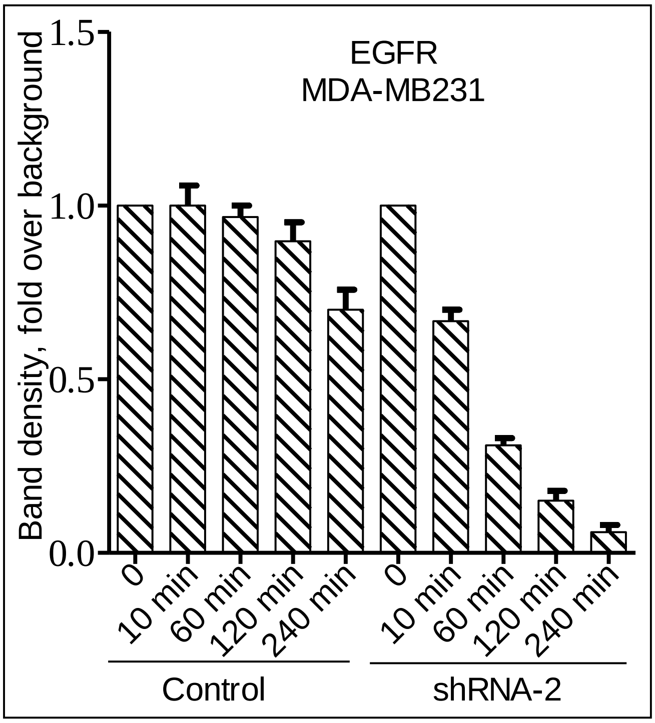

Supplementary figure 3a and b: Dynamics of EGFR degradation in the MDA-MB231 cells in the presence and absence of SHP2**. a**) First, EGFR was stabilized by chloroquine treatment for 6 hours and then cells were stimulated with 10 ng/ml EGF for the indicated time points. Lysates prepared from these cells were analyzed by immunoblotting with anti-EGFR antibody. As shown, EGFR was degraded relatively rapidly in the SHP2-silenced cells, but not in the control. The anti-SHP2 and the anti-β-actin immunoblotting show efficient SHP2 silencing and comparable total protein loading, respectively. **b**) Band density measurement of the EGFR immunoblotting. As shown, approximately 75% of EGFR in the SHP2-silnced cells was degraded within 1 hour, but only 30% was degraded in the controls even at the 4 hour time point. Data shown is mean ± standard error from three independent experiments.

c

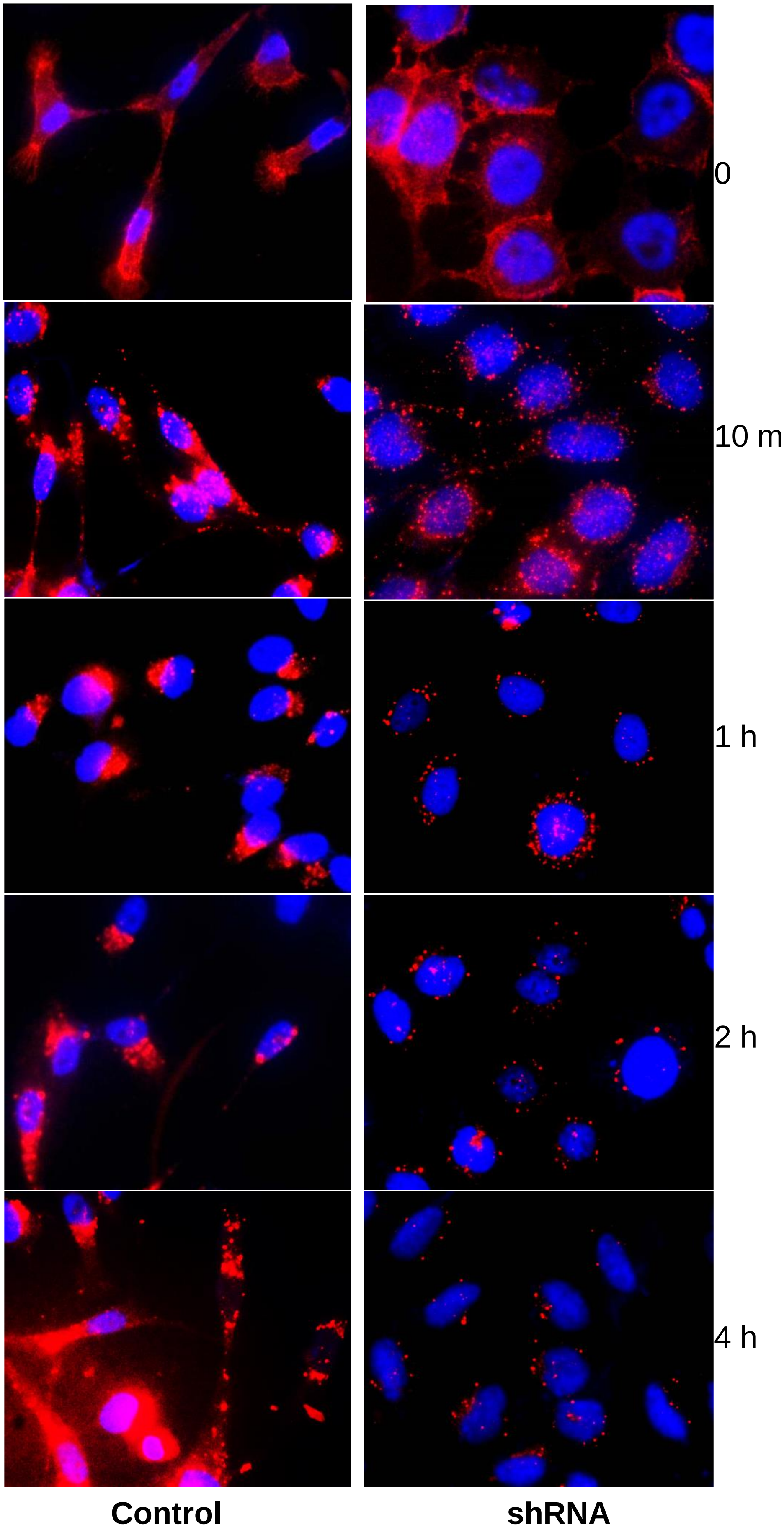

Supplementary figure 3c: Effect of SHP2 silencing on EGFR endocytosis and processing in the MDA-MB231 cells. EGF-induced EGFR endocytosis was not affected by SHP2 silencing, but the processing thereafter. While EGFR in the controls was sorted in polarized fashion with gradual outward extension, it remained perinuclear in the SHP2 silenced cells. In addition, it was possible to discern the rapid dissipation of the EGFR signal in the SHP2 silenced cells, but not in the controls.

D

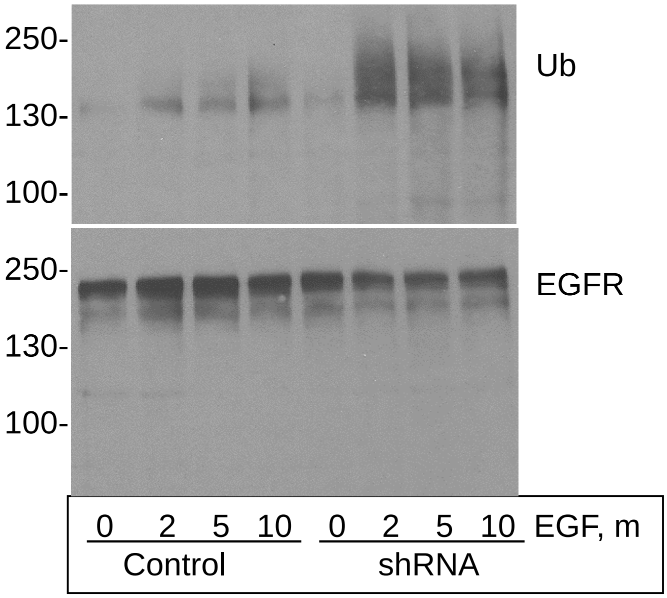

Supplementary figure 3D: Effect of SHP2 silencing on EGFR ubiquitination in the MDA-MB231 cells. First, EGFR was stabilized by chloroquine treatment for 6 hours and then cells were stimulated with 10 ng/ml EGF for the indicated time points. Lysates prepared from these cells were subjected to immunoprecipitation with anti-EGFR and immunoblotting with anti-ubiquitin antibodies. To compensate for the partial nature of EGFR restoration by chloroquine, the amount of EGFR precipitate loaded in the shRNA lanes was increased by 25%. As shown, EGFR was rapidly and highly ubiquitinated in the SHP2 silenced cells, but less so in the controls. Reprobing with anti-EGFR antibody showed comparable protein levels in all lanes.
