# Supplementary material for: SHP2 acts both upstream and downstream of multiple receptor tyrosine kinases to promote basal-like and triple-negative breast cancer
Source: Breast Cancer Res. 2016 Jan 4;18:2. doi: 10.1186/s13058-015-0659-z (PMC4700603; doi:10.1186/s13058-015-0659-z)
Supplement: Additional file 4: Figure S4. — EGFR and SHP2 IHC staining of a BTBC tumor. As shown, the tumor (Tum) is 3+ for both SHP2 and EGFR, while the corresponding normal (Nor) tissue is negative for both proteins. Immunofluorescent staining of a BTBC tumor section shows the expression of EGFR and SHP2, which was analyzed by costaining. As shown, the IF intensity was low in the normal region, medium in the hyperplastic region, and higher in the ductal carcinoma in situ (DCIS) and infiltrating ductal carcinoma (IDC) regions. (PDF 437 kb) [file 13058_2015_659_MOESM4_ESM.pdf]

A

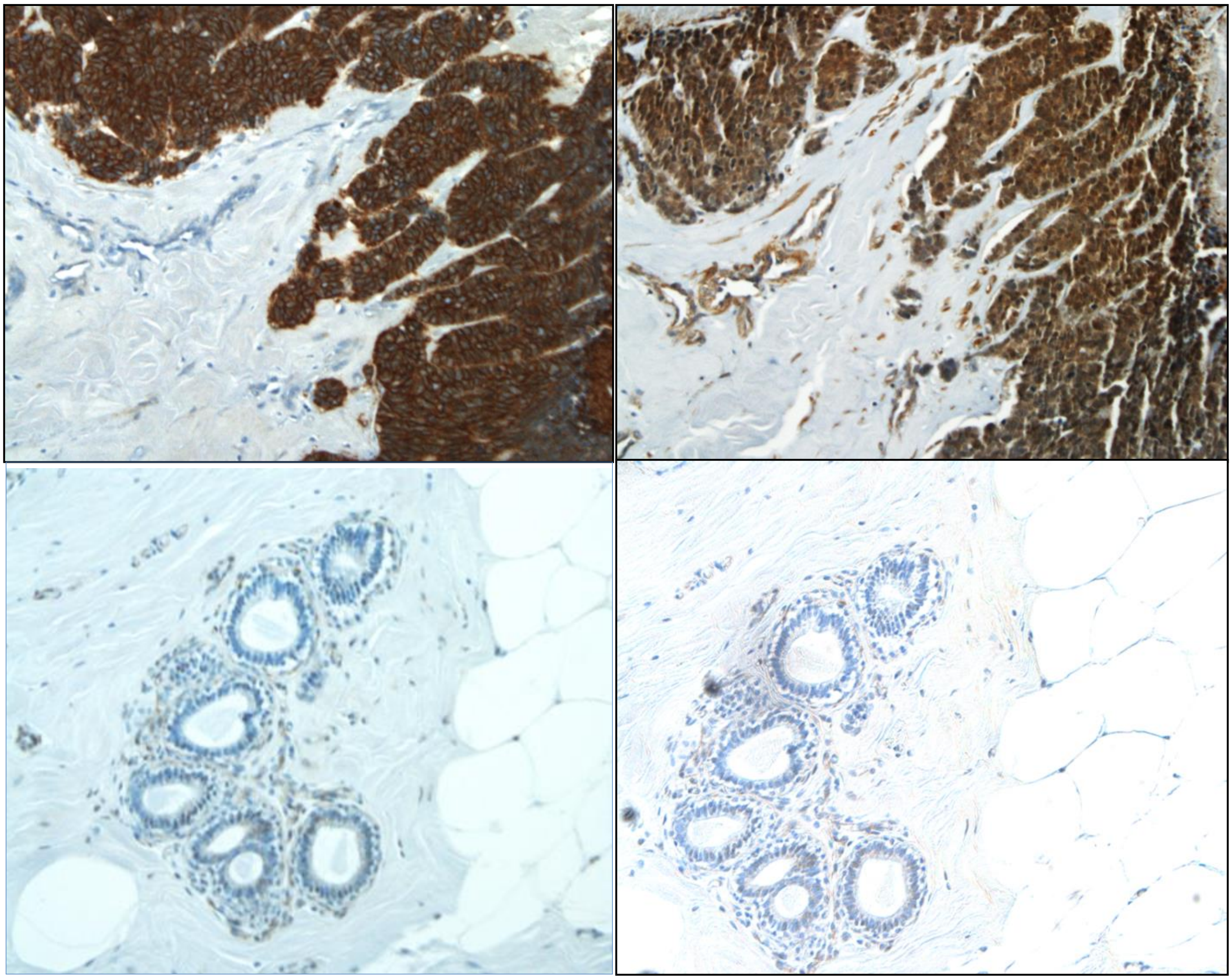

Supplementary Figure 4A: EGFR and SHP2 IHC staining of a BTBC case. As shown, the tumor (Tum) is 3+ for both SHP2 and EGFR, while the corresponding normal (Nor) tissue is negative for both proteins.

B

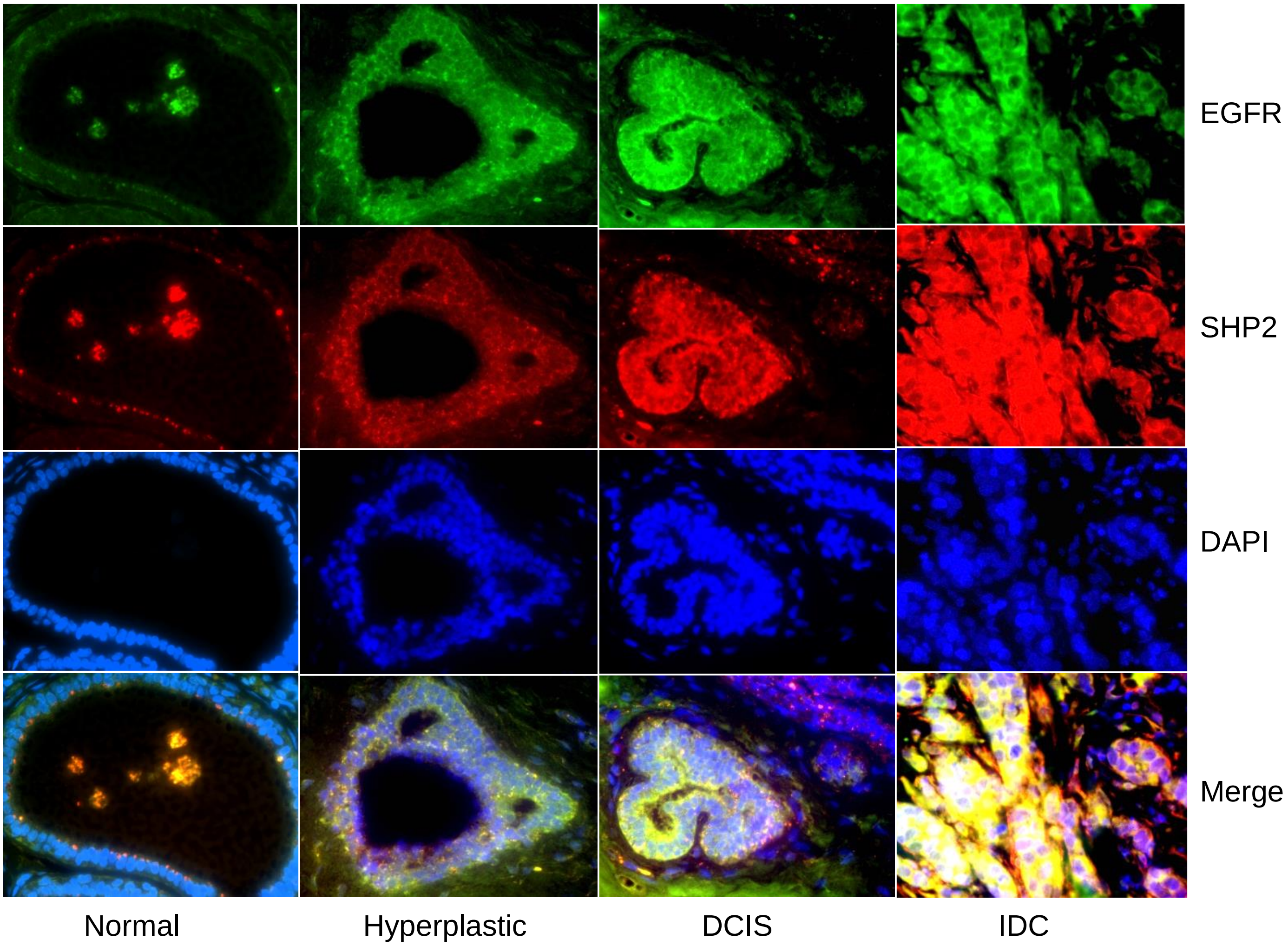

Supplementary Figure 4B: Immunofluorescence (IF) staining of a BTBC tumor section. The expression of EGFR and SHP2 was analyzed by co-staining. As shown, the IF intensity was low in the normal region, medium in the hyperplastic, and higher in the DCIS (ductal carcinoma *in situ*) and IDC (infiltrating ductal carcinoma) regions.
