# Supplementary material for: SHP2 acts both upstream and downstream of multiple receptor tyrosine kinases to promote basal-like and triple-negative breast cancer
Source: Breast Cancer Res. 2016 Jan 4;18:2. doi: 10.1186/s13058-015-0659-z (PMC4700603; doi:10.1186/s13058-015-0659-z)
Supplement: Additional file 5: Figure S5. — a Effect of SHP2 and EGFR silencing on primary and secondary mammosphere formation by the MDA-MB-468 cells. Note that the mammosphere-forming capacity of the SHP2-silenced cells was exhausted in secondary passaging, while a modest reduction was observed in the EGFR-silenced cells. b Effect of SHP2 and EGFR silencing on proportion of ALDH1-high cells. Silencing SHP2 expression drastically reduced the ALDH1-high cells, but silencing EGFR had only a modest effect. (PDF 237 kb) [file 13058_2015_659_MOESM5_ESM.pdf]

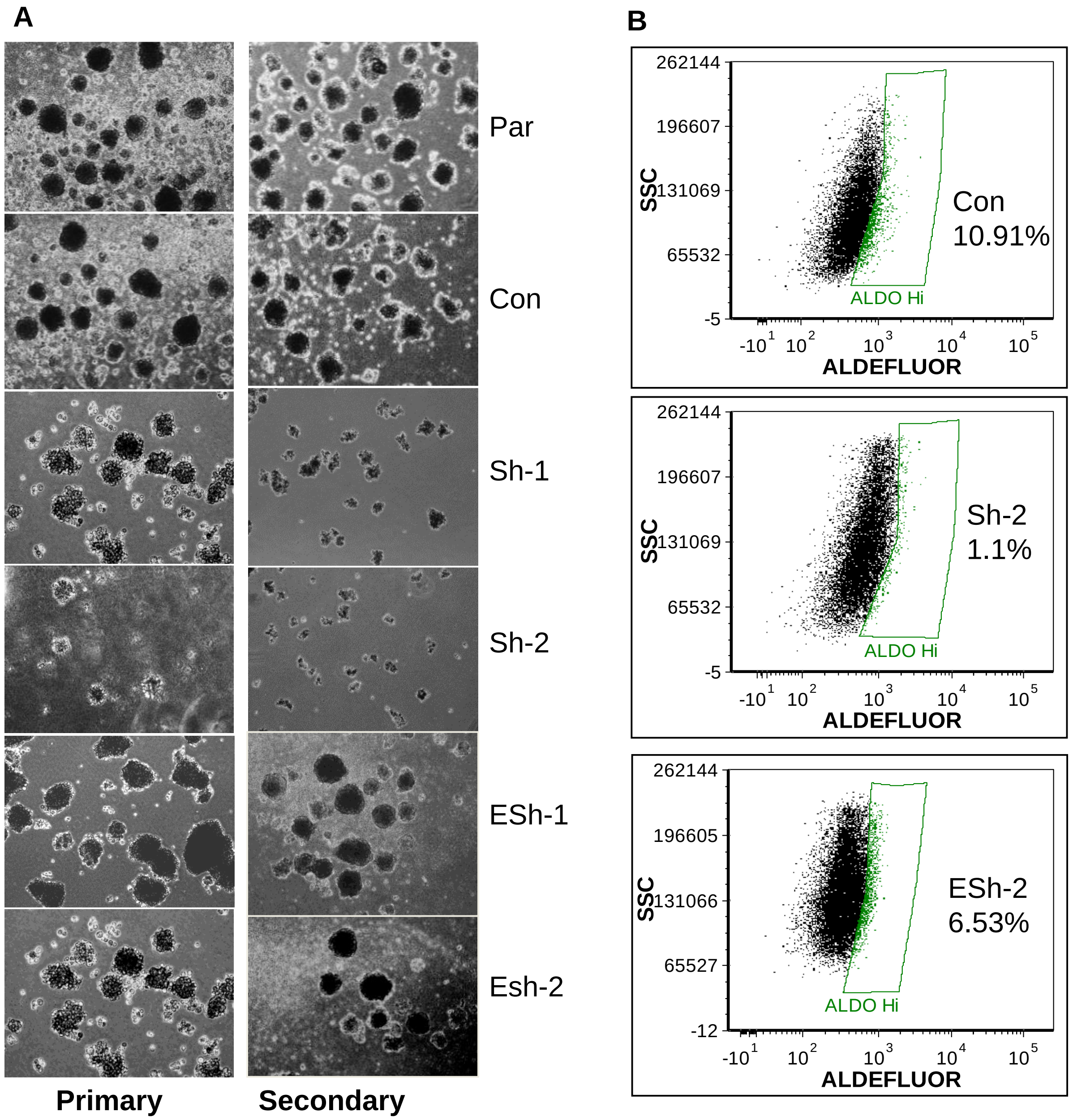

Supplementary Figure 5: A) Effect of SHP2 and EGFR silencing on primary and secondary mammosphere formation by the MDA-MB468 cells. Note that mammosphere-forming capacity of the SHP2-silenced cells was exhausted in secondary passaging, while a modest reduction was observed in the EGFR silenced cells. **B**) Effect of SHP2 and EGFR silencing on proportion of ALDH1-high cells. Silencing SHP2 expression drastically reduced the ALDH1-high cells, but silencing EGFR had only a modest effect.
