# Supplementary material for: SHP2 acts both upstream and downstream of multiple receptor tyrosine kinases to promote basal-like and triple-negative breast cancer
Source: Breast Cancer Res. 2016 Jan 4;18:2. doi: 10.1186/s13058-015-0659-z (PMC4700603; doi:10.1186/s13058-015-0659-z)
Supplement: Additional file 6: Table S1. — Internal codes for each case and the state of EGFR and SHP2 expression for each tumor. The first 12 cases were reanalyses of samples from a previous publication [14] in which the indicated samples were reported as triple-negative. Cases 84–127 are new BTBC samples. Yellow highlighting represents the co-overexpression of SHP2 and EGFR, cyan blue represents SHP2-positive tumors that do not have EGFR overexpression, and gray represents EGFR-positive tumors without SHP2 overexpression. (DOC 66 kb) [file 13058_2015_659_MOESM6_ESM.doc]

**Supplementary table 1:** In this table, the internal codes for each case and the state of EGFR and SHP2 expression for each tumor are provided. The first 12 cases were re-analyses of samples from our previous publication , in which the indicated samples were reported as triple-negative. Cases 84 – 127 are new BTBC samples. The yellow highlight shows co-overexpression of SHP2 and EGFR, the cyan blue shows SHP2-positive tumors that do not have EGFR overexpression, and the gray shows EGFR-positive tumors without SHP2 overexpression.

| Internal code | SHP2 score | EGFR score |
| --- | --- | --- |
| Case # 47 | 1+ | 0 |
| Case #50 | 2+ | 2+ |
| Case #56 | 0 | 0 |
| Case #58 | 2+ | 2+ |
| Case #59 | 1+ | 0 |
| Case #63 | 2+ | 2+ |
| Case #66 | 0 | 0 |
| Case #73 | 2+ | 1+ |
| Case #75 | 2+ | 0 |
| Case #76 | 0 | 0 |
| Case #78 | 0 | 0 |
| Case #81 | 1+ | 2+ |
| Case #84 | 2+ | 1+ |
| Case #85 | 1+ | 1+ |
| Case #86 | 3+ | 2+ |
| Case #87 | 2+ | 2+ |
| Case #88 | 2+ | 1+ |
| Case #89 | 3+ | 3+ |
| Case #90 | 3+ | 3+ |
| Case #91 | 2+ | 1+ |
| Case #92 | 3+ | 3+ |
| Case #93 | 3+ | 2+ |
| Case #94 | 3+ | 3+ |
| Case #95 | 2+ | 1+ |
| Case #96 | 3+ | 2+ |
| Case #97 | 3+ | 3+ |
| Case #98 | 3+ | 3+ |
| Case #99 | 3+ | 3+ |
| Case #100 | 1+ | 1+ |
| Case #101 | 1+ | 1+ |
| Case #102 | 1+ | 2+ |
| Case #103 | 2+ | 1+ |
| Case #104 | 1+ | 1+ |
| Case #105 | 2+ | 1 |
| Case #106 | 1+ | 0 |
| Case #107 | 3+ | 2+ |
| Case #108 | 2+ | 2+ |
| Case #109 | 3+ | 3+ |
| Case #110 | 3+ | 2+ |
| Case #111 | 1+ | 1+ |
| Case #112 | 2+ | 2+ |
| Case #113 | 2+ | 2+ |
| Case #114 | 0 | 0 |
| Case #115 | 0 | 1+ |
| Case #116 | 2+ | 2+ |
| Case #117 | 2+ | 2+ |
| Case #118 | 3+ | 2+ |
| Case #119 | 3+ | 2+ |
| Case #120 | 3+ | 3+ |
| Case #121 | 1+ | 1+ |
| Case #122 | 3+ | 3+ |
| Case #123 | 3+ | 2+ |
| Case #124 | 3+ | 2+ |
| Case #125 | 3+ | 3+ |
| Case #126 | 3+ | 3+ |
| Case #127 | 3+ | 3+ |
